# ER stress and subsequent activated calpain play a pivotal role in skeletal muscle wasting after severe burn injury

**DOI:** 10.1371/journal.pone.0186128

**Published:** 2017-10-13

**Authors:** Li Ma, Wanli Chu, Jiake Chai, Chuanan Shen, Dawei Li, Xiaoteng Wang

**Affiliations:** Department of Burn & Plastic Surgery, Burns Institute, First Hospital affiliated to General Hospital of the Chinese People's Liberation Army, Beijing, China; University of Louisville School of Medicine, UNITED STATES

## Abstract

Severe burns are typically followed by hypermetabolism characterized by significant muscle wasting, which causes considerable morbidity and mortality. The aim of the present study was to explore the underlying mechanisms of skeletal muscle damage/wasting post-burn. Rats were randomized to the sham, sham+4-phenylbutyrate (4-PBA, a pharmacological chaperone promoting endoplasmic reticulum (ER) folding/trafficking, commonly considered as an inhibitor of ER), burn (30% total body surface area), and burn+4-PBA groups; and sacrificed at 1, 4, 7, 14 days after the burn injury. Tibial anterior muscle was harvested for transmission electron microscopy, calcium imaging, gene expression and protein analysis of ER stress / ubiquitin-proteasome system / autophagy, and calpain activity measurement. The results showed that ER stress markers were increased in the burn group compared with the sham group, especially at post-burn days 4 and 7, which might consequently elevate cytoplasmic calcium concentration, promote calpain production as well as activation, and cause skeletal muscle damage/wasting of TA muscle after severe burn injury. Interestingly, treatment with 4-PBA prevented burn-induced ER swelling and altered protein expression of ER stress markers and calcium release, attenuating calpain activation and skeletal muscle damage/wasting after severe burn injury. Atrogin-1 and LC3-II/LC3-I ratio were also increased in the burn group compared with the sham group, while MuRF-1 remained unchanged; 4-PBA decreased atrogin-1 in the burn group. Taken together, these findings suggested that severe burn injury induces ER stress, which in turns causes calpain activation. ER stress and subsequent activated calpain play a critical role in skeletal muscle damage/wasting in burned rats.

## Introduction

The degree of a burn is determined by the percentage of total body surface area (TBSA) affected and TBSA >30% is considered major (severe) burn [1, 2]. Burn injury involves multiple organ system dysfunction and sepsis, creating a complex network of metabolic interactions, which include inflammation, immobilization and stress [3–5]. These changes are associated with significant health issues such as weakness or decreased skeletal muscle function, resulting in hypoventilation, difficulty in weaning off respirators, and decreased mobility, all of which lead to significantly morbidity and mortality, and seriously affect the prognosis and quality of life of burned patients [6–9]. Previous studies demonstrated that the pathophysiological response to burn injury alters the balance between skeletal muscle protein anabolism and catabolism [10]. This results in immediate loss of skeletal muscle mass [11–13]. Interestingly, it was shown that burn injury results in atrophy and myogenesis stimulation in muscle due to a host systemic response, although myogenesis does not compensate for burn-induced cell death [14, 15]. Despite this impressive wealth of knowledge, the exact mechanisms of skeletal muscle mass loss after burn are not fully understood, and further research is required to identify effective therapeutic targets.

Studies previously showed that severely burned patients have significant and persisting hepatic endoplasmic reticulum (ER) stress [16, 17]. The endoplasmic reticulum is a critical site for the synthesis and modification of proteins. Alteration of ER homeostasis may result in ER stress, which plays important roles in metabolism, inflammation, and tumorigenesis. ER stress is associated with several markers such as inositol-requiring enzyme-1 (IRE1), PKR-like ER kinase (PERK), activating transcription factor-6 (ATF6), X-box binding protein 1 (XBP1), CCAAT-enhancer-binding protein homologous protein (CHOP), and eukaryotic translation initiation factor 2 subunit alpha (eIF2α), which are involved to various degrees in multiple diseases [18, 19]. ER is also responsible for Ca^2+^ storage. The ER in skeletal muscle is termed sarcoplasmic reticulum (SR), and provides the feedback control required to balance the processes of calcium storage, release, and reuptake. Previous findings suggest that varying degrees of SR stress occur in senile amyotrophy, inclusion body myositis, polymyositis, and other pathophysiological processes [20]. Nevertheless, few studies on ER stress in skeletal muscle post burn injury have been reported.

Autophagy is a process by which cells disassemble unnecessary or dysfunctional components. It is a natural response to environmental or metabolic stress and aims to cell survival under adverse conditions [21]. LC3-II is a protein that is recruited to autophagosome membrane and serve as a marker of autophagy [22]. The ubiquitin-proteasome system (UPS) is a process eliminating misfolded proteins and mediates some autophagic processes [23]. Under ER stress, the UPS is compromised, leading to an accumulation of misfolded proteins and vulnerability of cells to adverse conditions [24]. Autophagy, especially macroautophagy, then allows bulk recycling of misfolded proteins and other damaged organelles during ER stress [25]. Atrogin-1 and MuRF-1 are E3 ubiquitin ligases that are specifically expressed in skeletal muscles and proceed to the polyubiquination of proteins [26, 27].

Ca^2+^-dependent proteases are required for releasing actin and myosin from Z disk decomposition, which generates characteristic fragments of actin or other proteins degradable by ubiquitin proteases [28–30]. Interestingly, the ubiquitin proteasome pathway increases significantly after severe burns and sepsis [31]. Our previous studies indicated that the main contribution to skeletal muscle wasting after burn injury is an accelerated breakdown of myofibril proteins caused by the ubiquitin proteasome pathway [32, 33]. Ca^2+^-dependent proteases also play essential roles in skeletal muscle wasting. Calpains are Ca^2+^-dependent cysteine proteases found in all vertebrate cells [34]. Abnormally enhanced calpain activation is commonly observed in atrophic conditions like disuse, denervation, glucocorticoid treatment, and sepsis [35, 36]. Interestingly, calpain levels were found to be significantly increased after burn, with calpastatin (a calpain inhibitor) displaying low levels, suggesting that calpain inhibitors could be used for burn injury treatment [37].

Therefore, we hypothesized that ER stress correlates with calpain activation after burn, which may trigger profound protein degradation in skeletal muscle, constituting the pathological basis of skeletal muscle wasting. We aimed to explore and confirm these underlying mechanisms of skeletal muscle damage/wasting post-burn using 4-phenylbutyrate (4-PBA), which is a chemical chaperone promoting protein folding and trafficking in ER and can be used as an inhibitor of ER stress [38].

## Methods

### Animals and grouping

All animal procedures conformed to the Guide for Care and Use of Laboratory Animals by the Chinese Academy of Science, and were approved by the Committee of Science and Technology of the First Hospital Affiliated to General Hospital of PLA (which is composed of scientists, veterinarians, ethics specialists, lawyer, and representatives of the general population, S1 Fig). Adult male *Wistar* rats (purchased from Chinese Medical Scientific Institute, Beijing, China) weighing 200–250 g were housed in wire bottom cages with a 12-h light-dark cycle. They were acclimated for seven days before the experiments, and received water *ad libitum* for the entire study period.

The animals were randomly divided into the sham, sham+4-PBA, burn, and burn+4-PBA groups (n = 32/group). All rats received general anesthesia (ketamine 40 mg/kg and xylazine 5 mg/kg, both injected intraperitoneally) prior to the modeling of burn injury (or sham burns). The burn and burn+4-PBA group animals received a full thickness thermal injury of 30% of TBSA by immersing the back of the trunk (12 seconds) and abdomen (6 seconds) in 94°C water, according to a previously described protocol [49]. The sham group was given sham burns by immersion in water at room temperature. After thermal or sham injury, the burned or sham-burned areas were dressed with 1% iodine tincture (Tianjin Pharmaceutical Group Co., Ltd., Tianjin, China), and the animals were immediately resuscitated with intraperitoneal Ringer's Lactate (40 ml/kg i.p.).

The burn+4-PBA and sham+4-PBA groups were treated with 4-PBA (Sigma Technology, USA) in 0.6% carboxymethylcellulose-Na (CMC-Na) at 700 mg/kg·d, i.g., from 1 to 14 days post burn; meanwhile, the other groups received equal amounts of CMC-Na.

The animals were monitored twice a day after burn injury. Rats were sacrificed at 1, 4, 7, and 14 days post burn by cervical dislocation. Then, TAM were excised, cleared of visible fascia, weighed, and examined by electron microscopy or stored in liquid nitrogen for other experiments.

### Transmission electron microscopy (TEM)

The TAM (n = 6 per group) specimens used for electron microscopy were initially kept in a slightly stretched state, using pins passed through tendons into dental wax, while they were fixed for 1 h in 2.5% glutaraldehyde in 0.2 M sodium cacodylate buffer (pH 7.2). Three muscle pieces (2×2×3 mm) were then excised and placed in fresh fixative for several hours. They were then post-fixed in 1% osmium tetroxide for 1 h, dehydrated in graded alcohol followed by propylene oxide, and embedded in epoxy resin. Sections were cut at a thickness of 80 nm using an ultra-microtome, collected on uncoated grids, stained with uranyl acetate and lead citrate, and examined on a transmission electron microscope JEM 1200EX II (JEOL, Japan).

### X-ray spectrometer-scanning electron microscopy for detecting Ca2+ signals

To observe Ca^2+^ signals in micro areas of skeletal muscle cells, TAM (n = 5) specimens were immersed in 5% glutaraldehyde solution for 2 h, and rinsed three times with 7.5% sucrose containing 90 mmol/L potassium oxalate solution. The tissues were then placed into 1% osmium tetroxide and 2% potassium pyroantimonate for 1.5 h. The samples were dehydrated with acetone and embedded in EPON812. Unstained ultrathin tissue sections were transferred in copper and plated on carbon film (thickness: 1000 nm). Tissue sections were analyzed on an x-ray spectrometer-scanning electron microscope, with the following parameters: accelerating voltage, 30 kV; probe inclination, 15°; beam spot diameter, 0.4 μm; scanning area, 2 μm^2^; and counting time, 80 s. In the cytoplasm and SR of skeletal muscle cells, electron probe x-ray microanalysis (EPMA) of Ca^2+^ was performed under a magnification of 15,000×. Ca^2+^ counts per second (cps) and peak to background ratio (p/b) were measured in order to calculate the Ca^2+^ signal intensity. Then, Ca^2+^ concentration (unit: mM) in the measured area was obtained according to its linear relationship with Ca^2+^ signal intensity. The Ca^2+^ standard solution was used to determine the linear relationship. For each specimen, 10 different regions were selected for scan, five each from cytoplasm and SR.

### Total RNA extraction and real-time polymerase chain reaction (RT-PCR)

After grinding in liquid nitrogen, total RNA was isolated from TAM specimens (n = 6) using TRIzol (Invitrogen, Carlsbad, CA, USA), and purified with Array Grade Total RNA Isolation Kit (SuperArray, Frederick, MD, USA). Then, cDNA synthesis was performed with PrimeScript II RTase (Takara, Japan), using 2 μg of total RNA for each sample. RT-PCR was carried out on a Bio-Rad 7500 Fast System (Bio-Rad, Hercules, CA, USA) and an iScript One-Step RT-PCR Kit with SYBR Green (Bio-Rad, Hercules, CA, USA). Amplification was performed at 94°C for 2 min, followed by 30 cycles of 94°C for 20 s, 54°C for 20 s, and 72°C for 30 s, and a final extension at 80°C for 20 s. Opticon Monitor Software (Version 3.1; GeneWorks, Inc., USA) was used to determine threshold cycles (CT), and relative quantification was calculated by the 2^-ΔΔCT^ method.

The primers were:

*calpain-1*: *5’-GGGGTGAAGTGGAGTGAAAG-3*’ (sense) and *5’-TTAAGGGCGTCAGGTGTAAGG -3’* (anti-sense);

*calpain-2*: *5’-GAAATCGAGGCCAACATTGAAGA -3’* (sense) and *5’-CTCCAGCCAGCTGAGCAAAC -3’* (anti-sense);

*18S* rRNA (internal control): *5‘-GCCAACACAGTGCTGTCT-3’* (sense) and *5’-AGGAGCAATGATCTTGATCTT-3’* (anti-sense).

### Western blot

TAM specimens (100 mg; n = 6) were homogenized in ice cold lysis buffer containing 50 mmol/L Tris-HCl (pH 7.6), 150 mmol/L NaCl, 0.1% sodium dodecyl sulfate (SDS), 1 mmol/L dithiothreitol, and 1× complete protease inhibitor cocktail. After centrifugation of homogenates at 12,000g for 10 minutes at 4°C, the supernatants were collected. Protein concentrations were determined using the Bio-Rad protein assay (Bio-Rad, Hercules, California). Equal protein amounts were separated by SDS-polyacrylamide gel electrophoresis and transferred onto polyvinylidene difluoride membranes at 60 V, 250 mA for 3 h. Then, the membranes were blocked in blocking buffer (5% skim milk in 0.1% Tween 20-Tris-buffered saline) for 2 h and incubated with primary antibodies [rabbit antibodies anti-XBP1 at 1:1000, anti-ATF6 at 1:1500 and anti-atrogin-1 at 1:1000 (Abcam plc, USA); rabbit antibodies anti-GRP78 at 1:1000, anti-CHOP at 1:1000, anti-p-eIF2αat 1:1000, anti-eIF2α at 1:1000, anti-LC3-A/B at 1:1000, anti-calpain-1 at 1:1000 and anti-calpain-2 at 1:1000 (Cell Signaling Technology, USA); rabbit antibodies anti-MuRF1 at 1:1500 and anti-GAPDH at 1:2000 (Santa Cruz Biotechnology, USA)] overnight at 4°C. After three washes, the membranes were incubated with horseradish peroxidase-conjugated goat anti-rabbit secondary antibodies 1:2000 (Zhongshan Golden bridge Biotechnology, Co, Ltd, China). Blots were developed with chemiluminescence substrate (ECL) (GE Healthcare Life Sciences, Piscataway, NJ) by autoradiography (Kodak Medical X-Ray Processor 102). Protein bands were analyzed using the Image J software, with protein expression levels expressed as the gray value ratio of target protein to GAPDH.

### Calpain activity

Calpain activity assays were performed using a commercially available kit (Biovision, Mountain View, CA, US), according to the manufacturer’s instruction. Briefly, 0.15 g of TAM specimens (n = 6) were minced on ice and mixed with ice-cold saline to a total volume of 1.5 mL. After homogenization on ice, equal amounts of protein lysate were mixed with fluorescence-labeled substrate for fluorometric detection on a microplate reader (SpectraMax M5, Molecular Devices, Sunnyvale, CA, USA).

### Statistical analysis

Statistical analyses were performed with the SPSS statistical software 22.0 (IBM, USA). Data are presented as mean ± standard error of the mean (SEM). Group comparisons were performed by the Student’s t test (between two groups) or one-way analysis of variance (ANOVA) (among ≥3 groups) with Tukey’s test for post-hoc analysis. *P*<0.05 was considered statistically significant.

## Results

### Severe burn injury induces myofibril damage and ER stress in tibialis anterior muscles (TAM)

**Fig 1:**
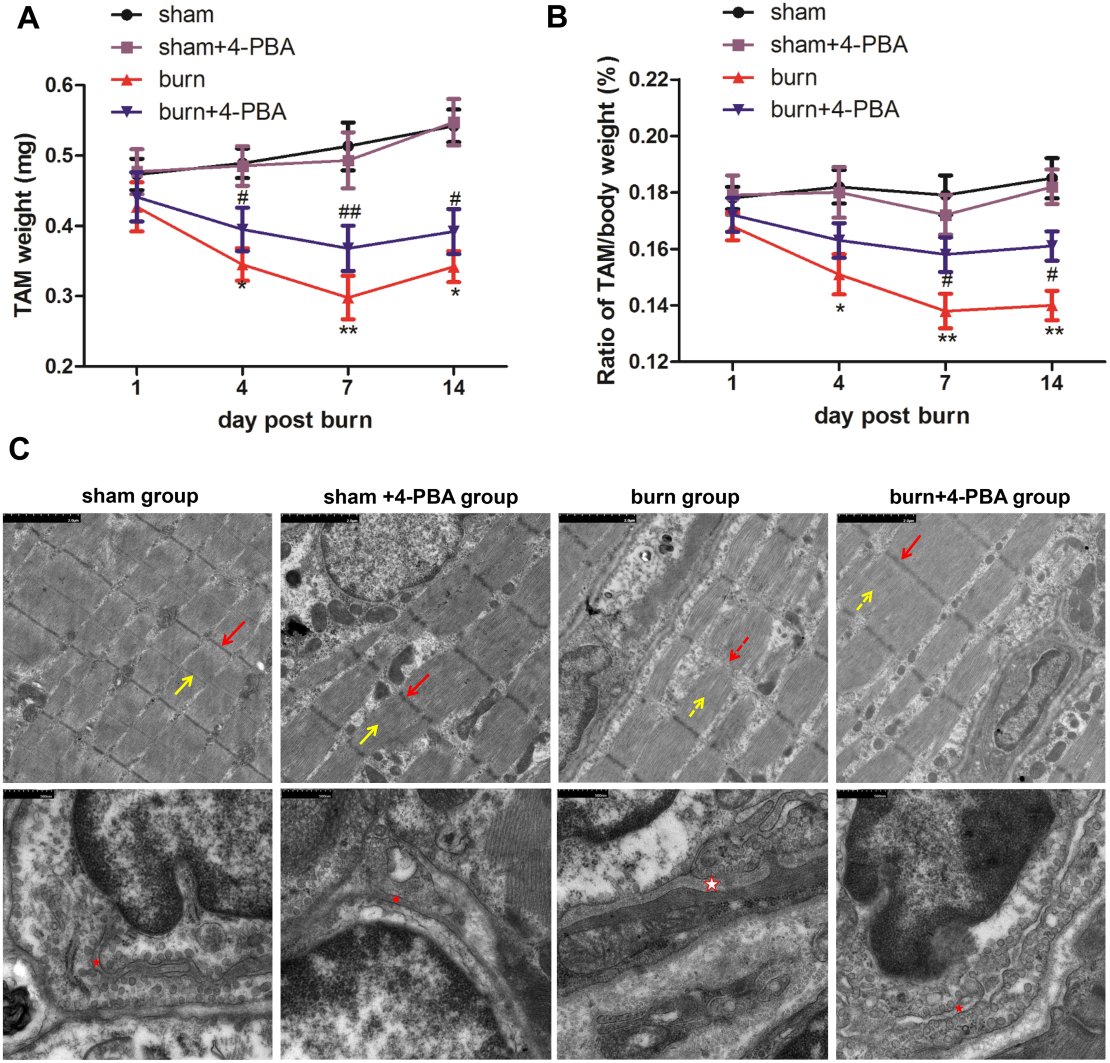
Severe burn injury induces myofibril damage and weight loss in tibialis anterior muscles, which could be alleviated by 4-PBA treatment. The rats were randomized to four groups: sham, sham+4-phenylbutyrate (4-PBA), burn and burn+4-PBA. Tibialis anterior muscle (TAM) weight (A) was measured and the ratio of TAM to body weight (B) was calculated at different time points post burn (or sham treatment). Ultrastructural changes at 7 days post-burn (C) were observed. Transmission electron microscope (TEM) images of TAM myofibrils (upper; scale bar: 2 μm) and TAM cells (bottom; scale bar: 500 nm) were shown. Z-line (red arrow), M-line (yellow arrow), and the sarcoplasmic reticulum (red asterisk) were represented. The dashed arrow indicates the broken structure, and the hollow asterisk indicates swollen structure. n = 6 per group. *p<0.05, ** *p*<0.01 vs. sham group; #*p*<0.05, ## *p*<0.01 vs. burn group.

Within 72 h after thermal injury (or sham burn), 0, 0, 7, and 4 rats died in the sham, sham+4-BPA, burn, and burn+4-PBA groups, respectively. Body weight loss (S2 Fig) was observed in the burn group compared with sham animals, accompanied by TAM weight reduction (Fig 1A) and decreased TAM to body weight ratio (Fig 1B). This trend became increasingly pronounced till 7 days post burn, but reboFunded at 14 days post burn.

Next, the structure of TAM myofibril was compared between the burn and sham groups at 7 days post burn, when skeletal muscle wasting had become the most severe. In the sham group, myofibrils were whole, with regular sarcomeres, and the Z line, M line, and H band were clear (Fig 1C). Meanwhile, in the burn group, the Z-line was ruptured with degraded M-line, and I band was white. In addition, the TEM images of TAM cells showed that the SR was significantly swollen in the burn group compared to sham animals.

**Fig 2:**
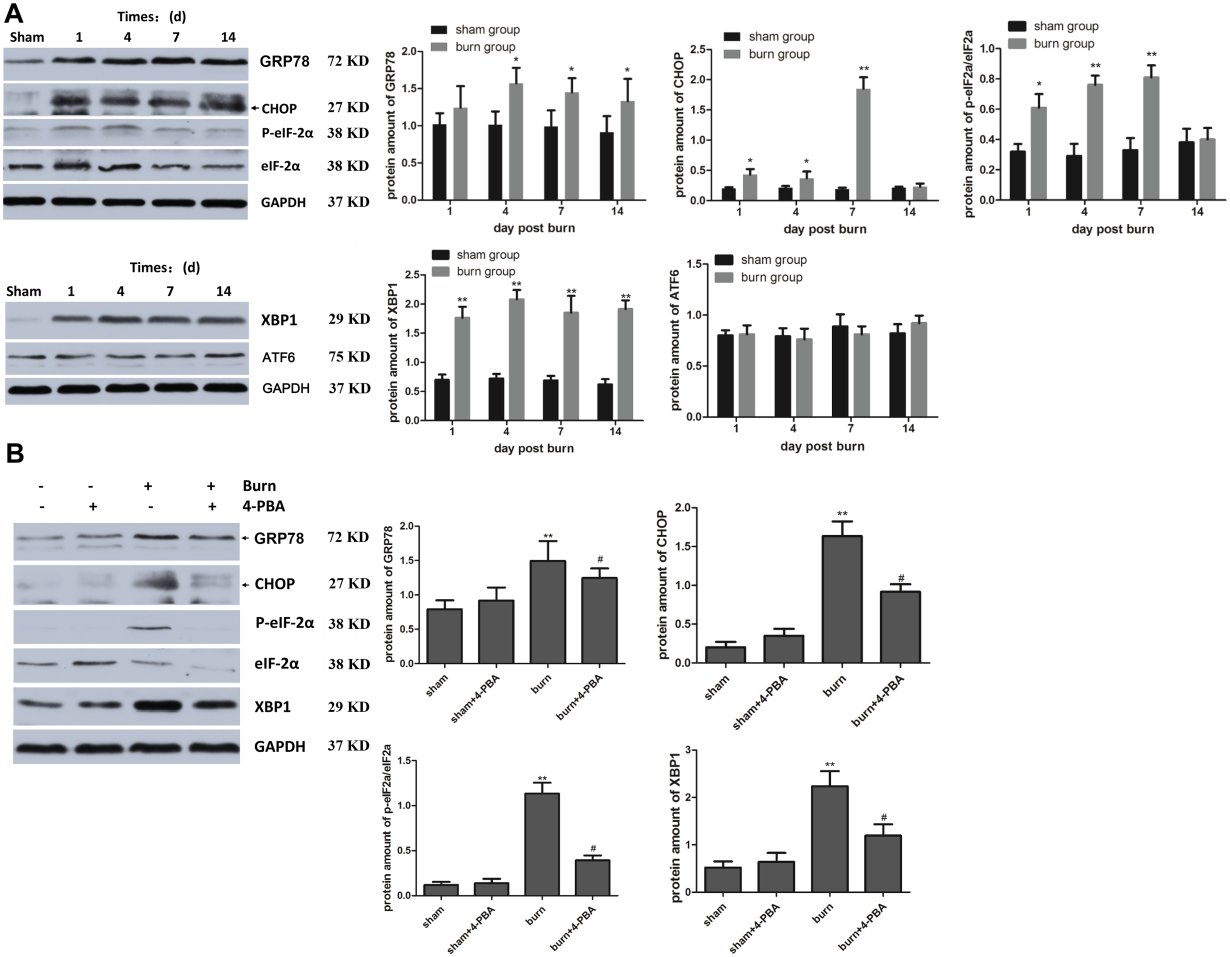
Severe burn injury induces ER stress in tibialis anterior muscles, which could be attenuated by 4-PBA treatment. The rats were randomized to four groups: sham, sham+4-PBA, burn and burn+4-PBA. Several markers of ER stress were assessed, including GRP78, CHOP, p-eIF2α, XBP1 and ATF6 in tibialis anterior muscle (TAM) specimens post-burn by western blot. Representative blots and statistical histograms showed the changes in various stress marker proteins at different time points after burn injury (A) and the relieving effects of 4-PBA at 7 days post-burn (B). The amount of proteins was quantified as normalized to the internal control GAPDH, except for p-EIF2α (using p-EIF2α/EIF2α ratio). n = 6 per group. *p<0.05, ** *p*<0.01 vs. sham group. # *p*<0.05 vs. burn group.

The morphological changes of the SR suggested that burn injury may induce ER stress in TAM. To test this hypothesis, we analyzed the amount of well-known ER stress markers in TAM. As shown in Fig 2A, the protein levels of GRP78, CHOP, p-eIF2α, and XBP1 changed in a time dependent manner (generally peaking at 7 days), while there were no differences in ATF6. These results confirmed that ER stress was increased in TAM after burn, suggesting one of the plausible mechanisms underlying the weight loss of TAM.

### Cellular Ca2+ homeostasis is significantly altered in TAM after severe burn injury

**Fig 3:**
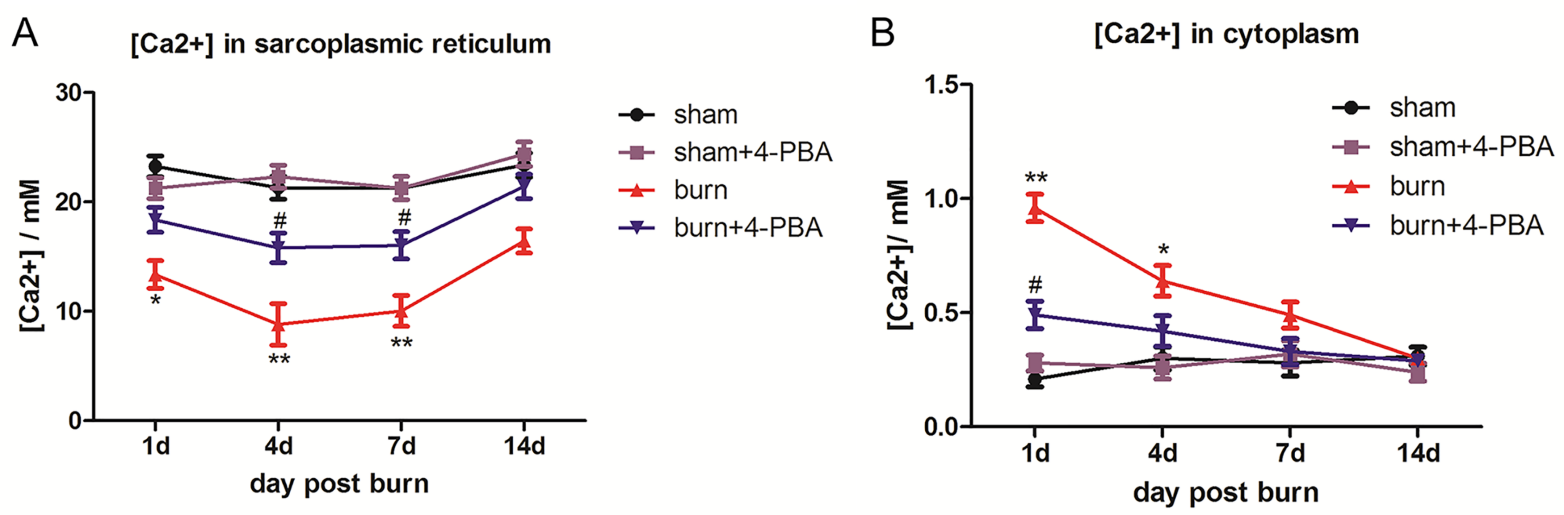
Severe burn injury induces changes in subcellular distribution of calcium in TAM cells, which could be reversed by 4-PBA treatment. The rats were randomized to four groups: sham, sham+4-PBA, burn and burn+4-PBA. Calcium concentration in the sarcoplasmic reticulum (A) and cytoplasm (B) of TAM cells at different time points post-burn are shown. n = 5 per group. *p<0.05, ** *p*<0.01 vs. sham group. #*p*<0.05 vs. burn group.

We further hypothesized that ER stress may influence the Ca^2+^ storage in SR. To test whether the release of Ca^2+^ from SR to cytoplasm was changed, the intracellular Ca^2+^ concentration were assessed in TAM cells of the burn and sham groups. Interestingly, SR Ca^2+^ levels were significantly lower in TAM cells in the burn group at 1, 4, and 7 days post burn (Fig 3A); meanwhile, cytoplasmic Ca^2+^ amounts were significantly elevated in the burn group at 1 and 4 days post burn (Fig 3B), compared with the sham group. These data suggested that cytoplasmic Ca^2+^ concentration may rise in TAM cells after burn injury resulting from a release of SR storage.

### Calpain is activated after severe burn injury

**Fig 4:**
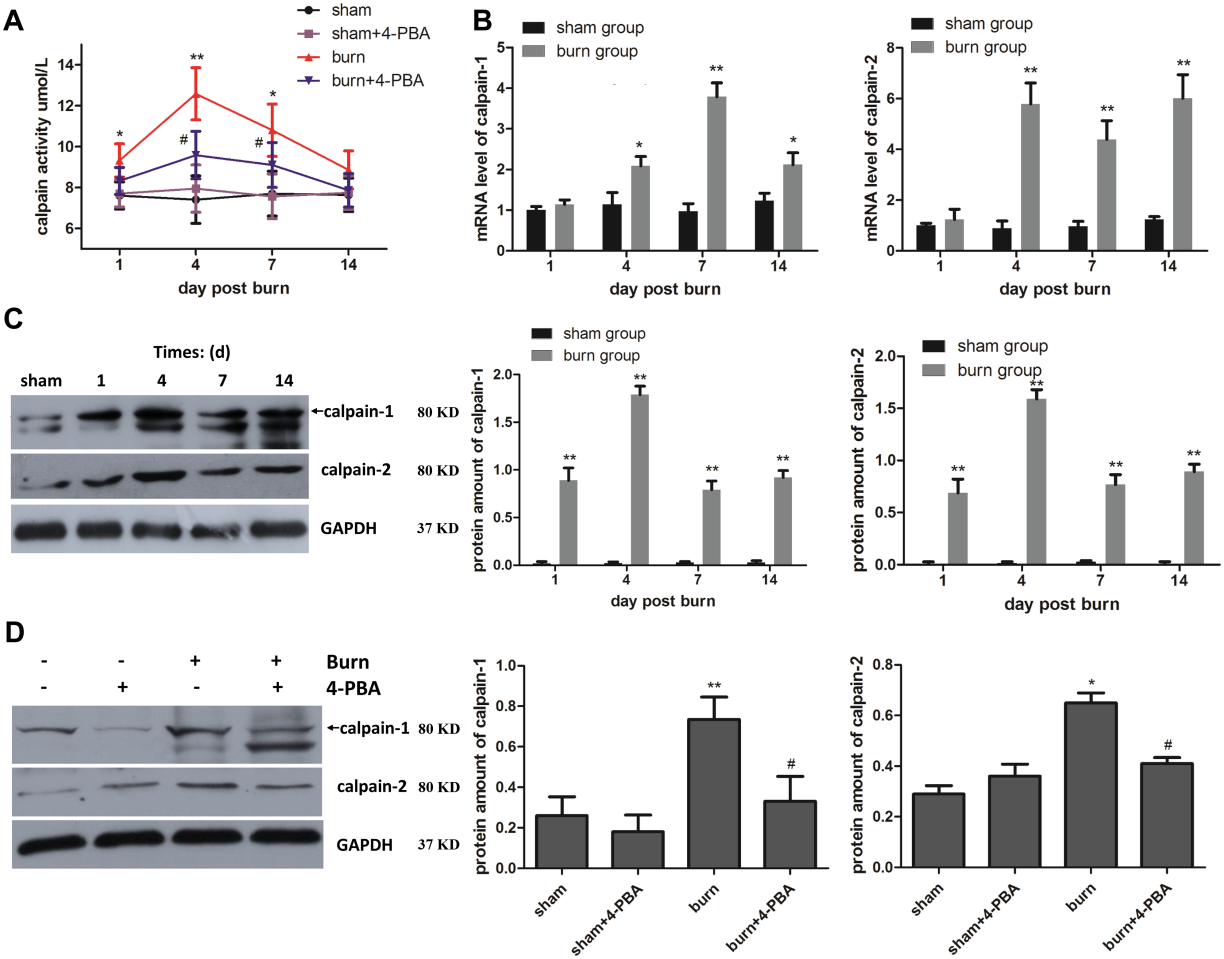
Calpain is overexpressed and activated after severe burn injury, which could be suppressed by 4-PBA treatment. The rats were randomized to four groups: sham, sham+4-PBA, burn and burn+4-PBA. Calpain activity was measured at different time points post-burn in TAM specimens (A). Gene expression levels and protein amounts of calpain-1 and 2 in TAM specimens were respectively evaluated by real-time PCR (B) and western blot (C), with GAPDH used as an internal control; the results were normalized to sham group. At 7 days post-burn, the effects of 4-PBA on the protein amounts of calpain-1 and 2 in TAM samples were examined by western blot (D). n = 6 per group. *p<0.05, ** *p*<0.01 vs. sham group. #*p*<0.05 vs. burn group.

The Ca2+ flow from SR storage to the cytoplasm may activate Ca^2+^-dependent cysteine proteases, growth factors, and hormones. Indeed, compared with sham group, calpain activation was enhanced post burn (Fig 4A). In addition, calpain-1 and calpain-2 levels were significantly increased in TAM from the burn group compared with those from sham animals at 4, 7, and 14 days post burn, both at the mRNA (Fig 4B) and protein (Fig 4C) levels. Of note, changes of protein levels were detected earlier than those of gene expression, as early as 1 day post-burn.

### Cellular Ca2+ homeostasis alteration and calpain activation after severe burn injury are caused by ER stress in TAM cells

To confirm that the changes of cytoplasmic Ca^2+^ and calpain activity could be attributed to ER stress in TAM cells after severe burn injury, the animals were administered 4-PBA, a molecular chaperone for ER stress. Interestingly, treatment with 4-PBA markedly reduced the protein amounts of the stress markers GRP78, CHOP, p-eIF2α, and XBP1 in burn animals (Fig 2B). Meanwhile, Ca^2+^ homeostasis in SR and cytoplasm were also restored by 4-PBA (Fig 3). Finally, 4-PBA reversed the increased calpain activation (Fig 4A) as well as the increased protein levels (Fig 4D) and mRNA expressions (S3 Fig) of calpain-1 and calpain-2 observed after burn injury. There were no differences between the sham and the sham+4-PBA groups. Taken together, the results suggested that Ca^2+^ flow from SR to cytoplasm and consequent calpain activation in TAM cells were caused by ER stress upon severe burn injury.

### Attenuation of ER stress by 4-PBA treatment prevents skeletal muscle from damage induced by severe burn injury

The structural damage of TAM myofibrils was significantly alleviated in burned rats administered 4-PBA at 7 days post burn (Fig 1C). After 4-PBA treatment, TAM myofibrils were whole and Z-lines clear. Meanwhile, the SR showed less swelling than that in burned animals as a consequence of ER stress inhibition by 4-PBA. Furthermore, we found that 4-PBA treatment alleviated the loss of body weight (S1 Fig) and TAM weight (Fig 1A) from 7 days to 14 days post burn (*p*<0.05 or *p*<0.01), similar effects were observed on the TAM to body weight ratio (*p*<0.05; Fig 1B). There were no differences between the sham and the sham+4-PBA groups. These data indicated that ER stress attenuation by the molecular chaperone 4-PBA would decrease to some extent the structural damage and weight loss of TAM after severe burn injury, corroborating that ER stress was one of the crucial causes for the burn-induced skeletal muscle wasting.

### Severe burn injury also activates UPS and autophagy

**Fig 5:**
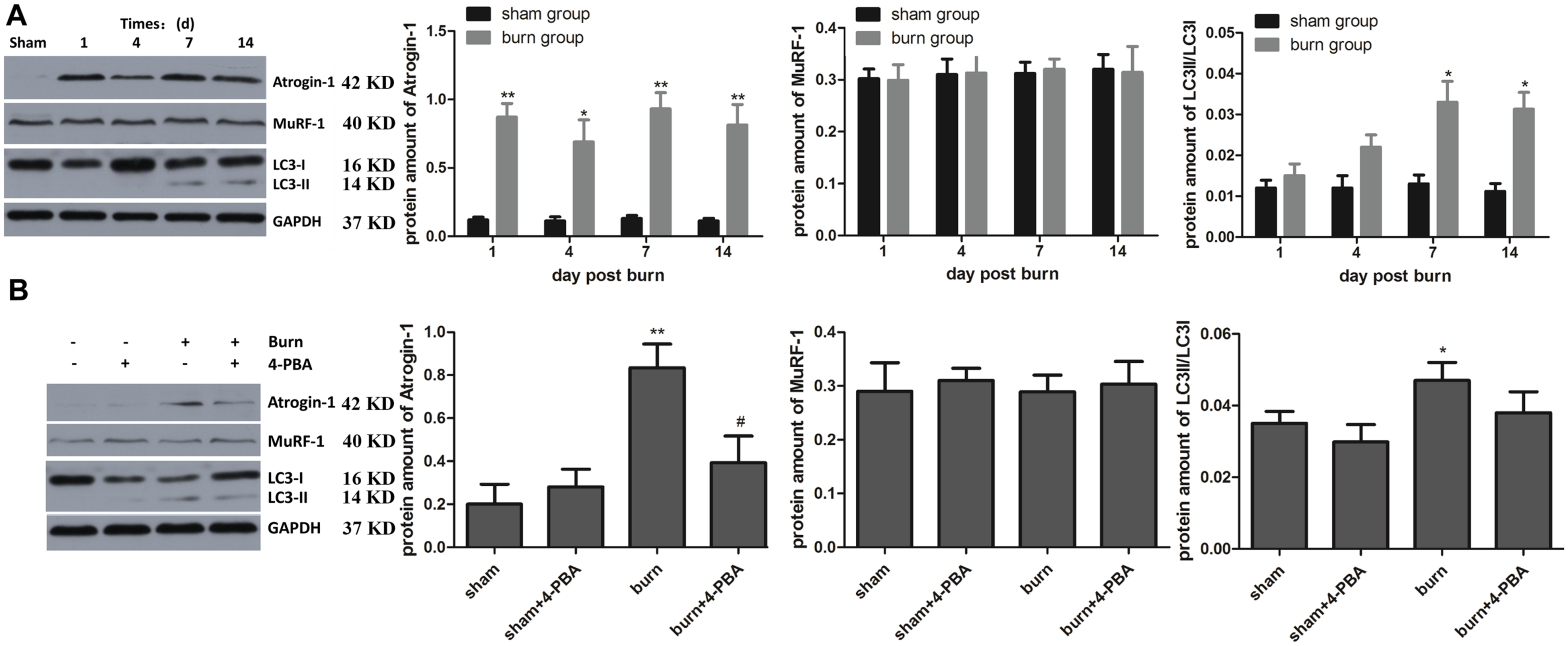
Severe burn injury activates UPS and autophagy, and the former may be inhibited by 4-PBA treatment to some extent. The rats were randomized to four groups: sham, sham+4-PBA, burn and burn+4-PBA. Markers of the muscle-specific ubiquitin-proteasome pathway (including atrogin-1 and MuRF1) and of autophagy (LC3-II/LC3-I ratio) were assessed in tibialis anterior muscle (TAM) specimens post-burn by western blot. Representative blots and statistical histograms showed the changes in various marker proteins at different time points after burn injury (A) and the relieving effects of 4-PBA at 7 days post-burn (B). The amount of proteins was quantified as normalized to the internal control GAPDH. n = 6 per group. *p<0.05, ** *p*<0.01 vs. sham group; # *p*<0.05 vs. burn group.

Other pathways reported to play important roles in wasting after severe burn injury were also examined, such as ubiquitin/proteasome and autophagy. Western blot showed that atrogin-1 was higher in the burn group than the sham group at all time points (*p*<0.05 or *p*<0.01), while burn had no effect on MuRF-1 (all *p*>0.05). The LC3-II/LC3-I ratio was elevated in the burn group at 7 and 14 days compared with the sham group (both *p*<0.05) (Fig 5A). 4-PBA treatment decreased atrogin-1 in the burn group but has no obvious effect on LC3I/II (Fig 5B). The results suggested that UPS and autophagy were also involved in the responses of skeletal muscles to severe burn injury, and the former may be partially attributed to ER stress.

## Discussion

In this report, using a severe burn injury rat model and the ER stress antagonist 4-PBA, we assessed ER stress and the subsequent activation of calpains in skeletal muscle and their impacts on post-burn skeletal muscle metabolism. We found that burn induced obvious ER stress and elevated cytoplasmic Ca^2+^ concentrations in skeletal muscle. This stress response led to calpain activation and, consequently, to augment skeletal muscle damage and muscle metabolic alteration. This is the first study to report ER stress and calpain activation in skeletal muscle tissue after severe burn injury.

Our previous studies demonstrated that severe burn injury induces enormous ER stress on the liver and skeletal muscle, contributing to hepatic dysfunction and skeletal muscle cell apoptosis [39]. Skeletal muscle ER stress response, in particular, may be a mechanism of skeletal muscle apoptosis, insulin signaling alterations, and skeletal muscle wasting post burn. In this study, four well-known ER stress markers (GRP78, CHOP, p-eIF2α, and XBP1) were dramatically increased in TAM from the burn group at days 4 and 7 after burn, while SR was significantly swollen in TAM, compared with sham animals. These findings indicated that severe burn injury induces persisting ER stress in skeletal muscle. In addition, ER stress consequently elevated cytoplasmic Ca^2+^ amounts in skeletal muscle cells post burn, corroborating previous reports assessing liver after burn injury [16, 17, 39]. Compared with the sham group, cytoplasmic Ca^2+^ was significantly elevated in skeletal muscle from burned animals, while SR Ca^2+^ levels were significantly lower. It is possible that the elevated Ca^2+^ concentrations found in skeletal muscle cytosol resulted from ER storage caused by burn injury induced ER stress.

In this study, significantly increased activation, transportation, and expression of calpains were observed in the skeletal muscle from burn animals compared with the sham group at days 4, 7, and 14 after burn. This is consistent with the notion that ER stress produces Ca^2+^ overload in cells, and may thus contribute to initiating calpain activation. Calpains are early mediators in the breakdown of sarcomeric proteins, promoting the release of myofilaments (including actin and myosin) that can then undergo degradation by the ubiquitin-proteasome system or autophagy pathway. Previous studies [40, 41] provided evidence for a role of Ca^2+^-dependent mechanisms (probably at least in part reflecting calpain activation) in muscle wasting. Ruptured Z-lines, degraded M-lines, and non-integral myofibrils are markers of muscle damage. Meanwhile, the muscle to body weight ratio is a marker of muscle wasting. Here, the structure of TAM myofibrils from burned animals as well as muscle to body weight ratio were assessed at day 7 after burn injury. In the burn group, Z-line rupture and M- line degradation were observed, while I band became white. In addition, lower TAM to body weight ratio at post burn day 7 was observed. These findings suggested that calpain activation causes disruption of the skeletal muscle sarcomere, and sequentially induces skeletal muscle damage/wasting after severe burn injury. Although numerous reports showed that increased expression and activity of the ubiquitin-proteasome proteolytic pathway, including a dramatic upregulation of the muscle-specific ubiquitin ligases atrogin-1 and MuRF1, play an essential role in sepsis-induced muscle wasting [42, 43], the present study did provide evidence that activation of the calpain system is an additional important mechanism of burn-induced muscle wasting. Nevertheless, the present study showed that atrogin-1 was elevated in burned animals at all time points, but not MuRF1, suggesting than multiple control pathways could be involved. In addition, autophagy (as revealed by the LC3-II/LC3-I ratio) was increased at days 7 and 14 in burned animals, suggesting that autophagy is not involved in the early phase of muscle wasting after burn. A previous study showed that autophagy was elevated in hepatocytes after burn injury, but autophagy was not examined in time [16]. This requires additional studies to grasp the minute UPS and autophagy mechanisms involved in burn injury.

To confirm that ER stress could cause calpain activation and subsequently lead to skeletal muscle damage/wasting, 4-PBA, a pharmacological chaperone that alleviates ER stress [44], was administered to animals after burn injury. After 7 days, 4-PBA markedly attenuated burn-induced high expression of ER stress markers as well as atrogin-1. Meanwhile, TEM observation indicated that 4-PBA treatment caused reduced ER expansion in TAM. These findings suggested that 4-PBA attenuates burn-induced ER stress response in skeletal muscle. In addition, we found significantly decreased cytoplasmic Ca^2+^ levels and calpain activation in 4-PBA treated rats compared with the burn group. These data indicated that ER stress inhibition after burn is consistent with decreased calpain activation. Furthermore, compared with the burn group, rats administered 4-PBA had a tendency of less skeletal muscle weight loss accompanied by decreased ultrastructure damage. Therefore, we concluded that ER stress in skeletal muscle tissues may play a pivotal role in skeletal muscle damage/wasting after severe burn injury; the related regulatory mechanisms may, at least in part, involve calpain activation.

The purpose of using 4-PBA in the present study was solely to reduce ER stress, as in previous studies [45, 46]. Indeed, Bohnert et al. [47] showed that 4-PBA decreased muscle mass in mice. Another study that assessed the effects of 4-PBA on different muscles (soleus, extensor digitorum longus, and diaphragm) showed that it produced an impressive increase of cross-sectional area for all fiber types in mice with muscle weakness and atrophy (due to an *I4895T* mutation in the *RyR1* gene), but it did not change the fiber size in wild-type mice [48]. The controversial conclusions among studies could be attributed to variations in drug administration (including, but not limited to, dose and duration). In addition, the different animals (*C57BL/6* mice vs. *Wistar* rats) used and the age of animal may also influence the outcomes. On the other hand, with regard to the effect of 4-PBA on pathological models (as for the paper by Bohnert et al, skeletal muscle wasting during cancer cachexia; in our study, skeletal muscle atrophy was due to severe burns), the distinct etiological and pathological conditions would make a great difference in the outcomes. In the present study, there were no differences between the sham and sham+4-PBA groups. Nevertheless, the possibility that 4-PBA influences muscle atrophy through multiple pathways should be explored in future studies.

The present study is not without limitations. The sample size was limited and no replicates were performed. In addition, only juvenile rats were used. Therefore, the observations might not be only muscle wasting but also lack of growth or failure to thrive. Additional studies are necessary to examine the impact of age on muscle wasting after burn injury. Overall, we discovered that burn injury induced ER stress response and elevated cytoplasmic Ca^2+^ amounts in skeletal muscle. Subsequently, calpains were activated after burn. These pathological processes may trigger and exacerbate skeletal muscle damage/wasting after severe burn injury. Our findings add an important mechanism of burn-induced muscle wasting, providing new insights in preserving skeletal muscle homeostasis after severe burn injury. This can help further develop a protective strategy for severe burn patients.

## Supporting information

S1 Fig Statement of animal rights. All the procedures in the animal experiments were reviewed and approved by Institutional Animal Care and Use Committee (IACUC) of First Hospital Affiliated to PLA General Hospital.
(JPG)

S2 Fig Body weight measurement. The rats were randomized to four groups: sham, sham+4-PBA, burn and burn+4-PBA. Body weight was measured in these groups at different time points post burn (or sham treatment). n = 6 per group. *p<0.05, ** *p*<0.01 vs. sham group; #*p*<0.05, ## *p*<0.01 vs. burn group.
(TIF)

S3 Fig Gene expressions of calpain-1 and 2 in TAM were decreased by 4-PBA upon burn injury. At 7 days post-burn, the gene expression levels of calpain-1 and 2 in TAM specimens were evaluated by real-time PCR, with GAPDH used as an internal control; the results were normalized to sham group. n = 6 per group. ** *p*<0.01 vs. sham group; # *p*<0.05 vs. burn group.
(TIF)
