# Supplementary figures and images for: ER stress and subsequent activated calpain play a pivotal role in skeletal muscle wasting after severe burn injury
Source: PLoS One. 2017 Oct 13;12(10):e0186128. doi: 10.1371/journal.pone.0186128 (PMC5640216; doi:10.1371/journal.pone.0186128)

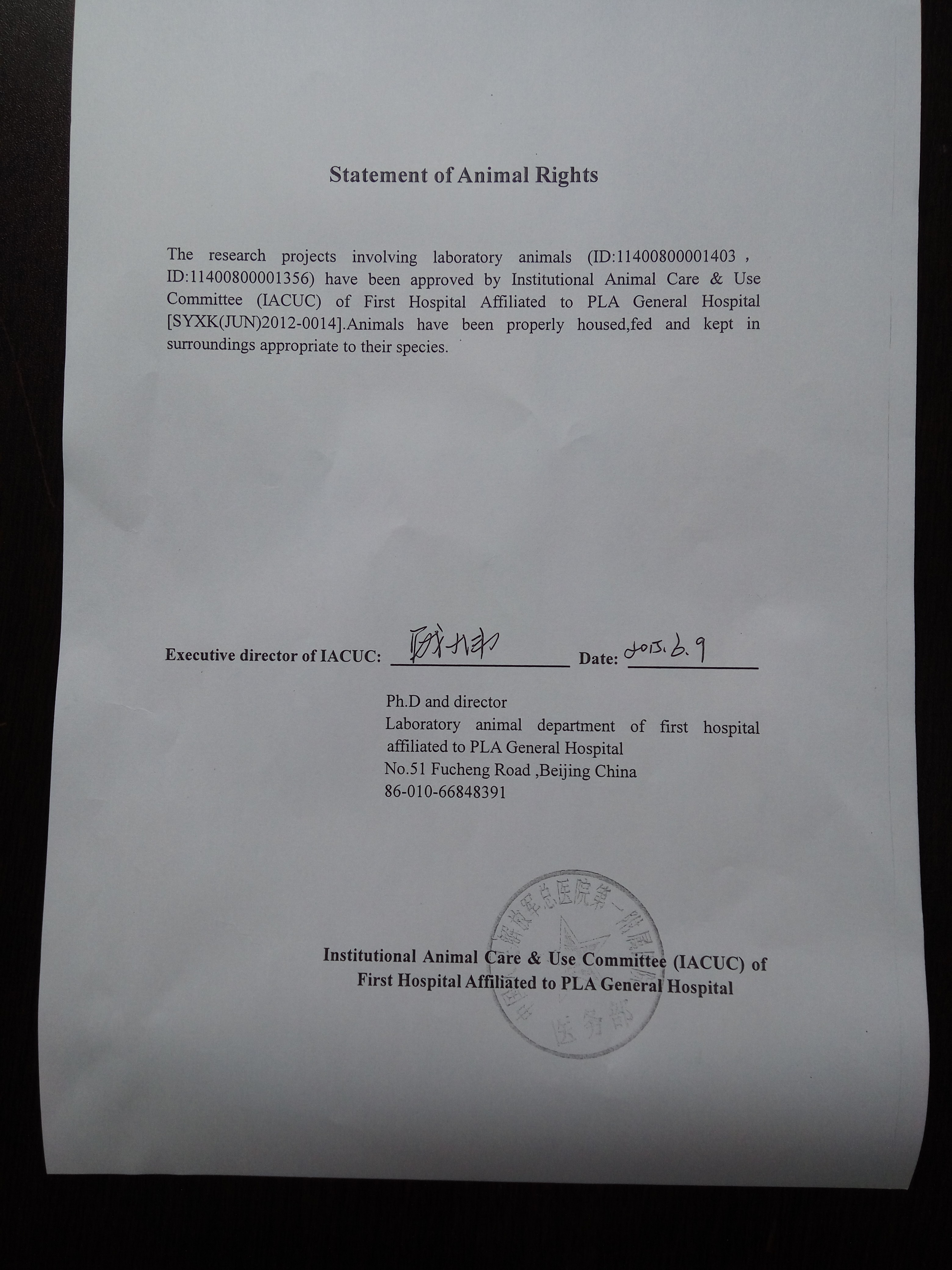

Supplement: S1 Fig — All the procedures in the animal experiments were reviewed and approved by Institutional Animal Care and Use Committee (IACUC) of First Hospital Affiliated to PLA General Hospital. (JPG) [file pone.0186128.s001.jpg]

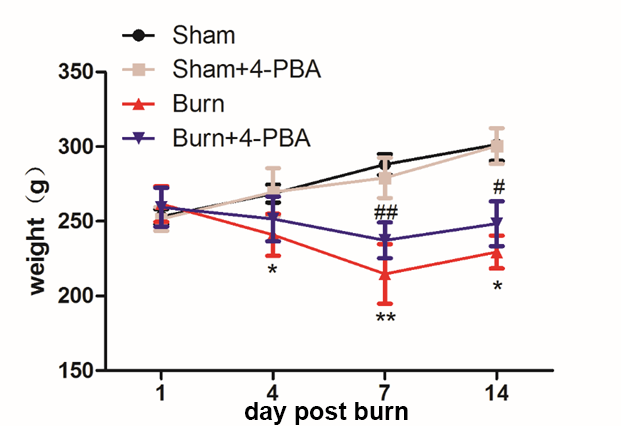

Supplement: S2 Fig — The rats were randomized to four groups: sham, sham+4-PBA, burn and burn+4-PBA. Body weight was measured in these groups at different time points post burn (or sham treatment). n = 6 per group. *p<0.05, ** p<0.01 vs. sham group; #p<0.05, ## p<0.01 vs. burn group. (TIF) [file pone.0186128.s002.tif]

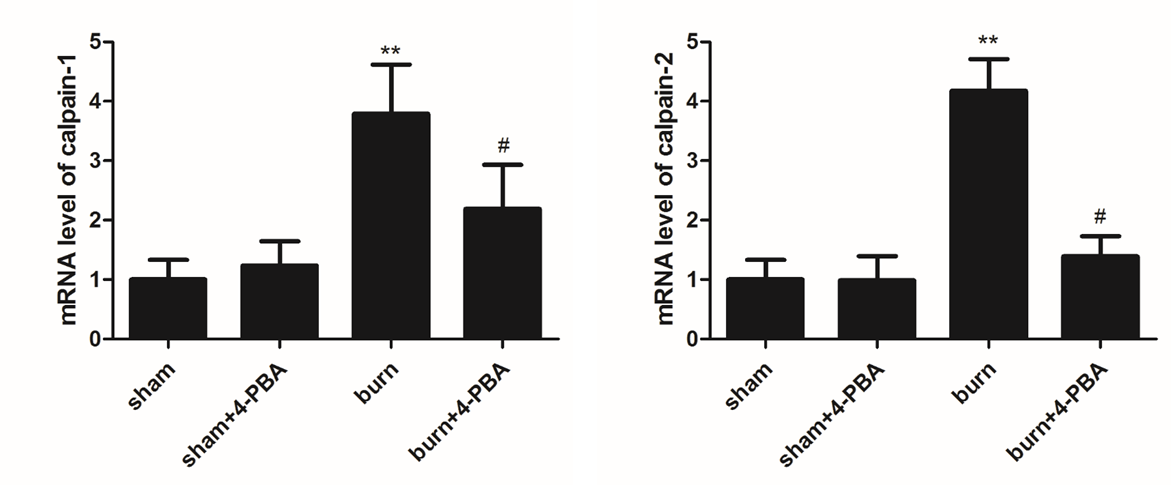

Supplement: S3 Fig — At 7 days post-burn, the gene expression levels of calpain-1 and 2 in TAM specimens were evaluated by real-time PCR, with GAPDH used as an internal control; the results were normalized to sham group. n = 6 per group. ** p<0.01 vs. sham group; # p<0.05 vs. burn group. (TIF) [file pone.0186128.s003.tif]
